# Eculizumab is efficacious and safe in pediatric patients with various forms of hemolytic uremic syndrome: a retrospective clinical experience of a tertiary center

**DOI:** 10.3389/fphar.2025.1535407

**Published:** 2025-04-04

**Authors:** Naama Lax, Miriam Davidovits, Gabriel Chodick, Yael Bernfeld, Orit Peled

**Affiliations:** ^1^ Department of Pharmacy, Schneider Children’s Medical Center of Israel, Petah Tikva, Israel; ^2^ Institute of Nephrology, Schneider Children’s Medical Center of Israel, Petah Tikva, Israel; ^3^ Faculty of Medicine, Tel-Aviv University, Tel-Aviv, Israel; ^4^ Maccabi Institute for Research and Innovation, Maccabi Healthcare Services, Tel Aviv, Israel; ^5^ Adelson School of Medicine, Ariel University, Ariel, Israel

**Keywords:** eculizumab, hemolytic uremic syndrome, pediatrics, aHUS, STEC-HUS, TA-TMA

## Abstract

**Background:**

Eculizumab, a terminal complement inhibitor, prevents thrombotic microangiopathy (TMA) and multiorgan damage in hemolytic uremic syndrome (HUS). We evaluated its efficacy and safety in pediatric patients with TMA sub-types: atypical HUS (aHUS), Shiga toxin-producing *Escherichia coli* (STEC)-HUS, and transplant-associated TMA (TA-TMA).

**Methods:**

This retrospective study included all pediatric patients treated with eculizumab for HUS at Schneider Children’s Medical Center (2011–2020), including those with pre-existing end-stage kidney disease. Clinical and laboratory parameters were analyzed over 28 weeks. The primary endpoint was achievement of complete TMA response, defined by sustained normalization of hematologic parameters and renal function. Secondary endpoints included TMA event-free status and additional clinical improvements.

**Results:**

Twenty-four pediatric patients (median age 5.8 years) were included: 13 with aHUS, 5 with STEC-HUS, and 6 with TA-TMA. A complete TMA response was achieved in 12 (50%) of the patients overall: 7 (54%) with aHUS, 3 (60%) with STEC-HUS, and 2 (33%) with TA-TMA. TMA event-free status was reached in 15 (63%) patients. Significant improvements were observed in platelet count (63%), lactate dehydrogenase levels (76% within the first week), hemoglobin (60%), and estimated glomerular filtration rate (79%); while CH-50 levels decreased. No severe adverse events were attributed to eculizumab. Chronic kidney disease stage improved for 17 (90%).

**Conclusion:**

The efficacy and safety of eculizumab for three TMA subtypes in pediatric patients potentially expands its therapeutic applications. The complete TMA response rate in aHUS supports eculizumab as a first-line use, while the response rate in STEC-HUS suggests potential efficacy beyond eculizumab’s primary indication. The early hematologic responses and reduced CH-50 levels confirm the role of eculizumab complement-mediated HUS and underscore the need for further research in TA-TMA.

## Introduction

Hemolytic uremic syndrome (HUS) is a group of diseases characterized by the triad of nonimmune microangiopathic hemolytic anemia, thrombocytopenia, and decreased renal function. HUS is the common clinical result of various pathological processes. Primary injury to the endothelium (mainly renal) causes cell swelling and detachment, formation of microthrombi, mechanical hemolytic anemia, and organ dysfunction, mainly of the kidneys. Extrarenal organ damage (i.e., neurological, cardiovascular, pulmonary, and gastrointestinal) sometimes also presents (Alfandary et al., 2020).

In its typical form, HUS is preceded by bloody diarrhea induced by a Shiga toxin (STX)-producing *Escherichia coli* (STEC), and transmitted by contaminated food or water (Monet-Didailler et al., 2020). Atypical HUS (aHUS) is a syndrome caused by inherited or acquired defects in regulation of the complement system’s alternative pathway, now described as “complement HUS”. Both dysregulation and excessive uncontrolled activation of the complement alternative pathway (Noris and Remuzzi, 2009; Zipfel et al., 2010; Zipfel and Skerka, 2009) result in persistent cleavage of complement protein C5 and formation of membrane attack complex (MAC). This leads to endothelial injury and systemic thrombotic microangiopathy (TMA) (Benz and Amann, 2010; Noris et al., 2010; Loirat and Frémeaux-Bacchi, 2011; Licht et al., 2015). Activation of the complement system is triggered by abnormal function of complement regulators: factor H, factor I, membrane cofactor protein, C3, and factor B. Genetic mutations have been identified in 50%–70% of patients with aHUS (Noris et al., 2010; Kavanagh and Goodship, 2011; Kavanagh and Goodship, 2010).

The various types of HUS are caused by different primary triggers; yet, their clinical features overlap (Alfandary et al., 2020). Several studies have demonstrated uncontrolled activation of the complement system in patients with STEC-HUS, as well as in patients after hematopoietic stem cell transplantation (HSCT), thus leading to transplant-associated TMA (TA-TMA) (Walsh and Johnson, 2019; Buelli et al., 2019; Poolpol et al., 2014; Orth et al., 2009; Dvorak et al., 2019; Cugno et al., 2021).

The classification of TA-TMA has evolved over recent years from traditionally categorized under secondary aHUS (Aklilu and Shirali, 2023; Goodship et al., 2017; Timmermans and van Paassen, 2021). Supported by literature and expert consensus, TA-TMA has increasingly classified as a distinct entity under the broader umbrella of TMA rather than as a subtype of secondary aHUS (Li and Sartain, 2024; Lazana, 2023). This shift reflects a growing understanding of its unique pathophysiology, particularly its strong association with endothelial injury rather than primary complement dysregulation.

Generally, aHUS has been treated with plasmapheresis, to induce stabilization of hematologic parameters, yet the efficacy has been limited. In observational studies, plasmapheresis was shown not to efficiently prevent the progression of tissue damage and substantial morbidity and mortality (Greenbaum et al., 2016; Fremeaux-Bacchi et al., 2013). This is due to the persistence of the underlying complement-mediated pathogenic mechanism of TMA (Noris et al., 2010; Fremeaux-Bacchi et al., 2013).

Eculizumab (Soliris^®^, Alexion Pharmaceuticals), a terminal complement inhibitor, is a recombinant, humanized, monoclonal immunoglobulin G antibody that targets human C5 complement protein and inhibits the subsequent formation of terminal MAC. Consequently, eculizumab is expected to prevent host cell damage as well as TMA-characterized triad and multiorgan damage (Loirat and Frémeaux-Bacchi, 2011), as manifested in complement HUS (Licht et al., 2015). Likewise in TA-TMA, in which complement system overactivation has been identified (Jodele et al., 2014), a blockade with eculizumab might be a suitable therapeutic strategy. Additionally, the demonstration of hyperactivation of complement by Shiga toxin in STEC-HUS raises the possibility that eculizumab may also have beneficial effects in typical-HUS (Noris et al., 2012; Karpman and Tati, 2016).

Retrospective and prospective clinical trials have established the efficacy and safety of eculizumab in adults with aHUS leading to its approval for pediatric patients with this condition (Rathbone et al., 2013; Legendre et al., 2013). Its beneficial effects in children with aHUS are supported by prospective and retrospective studies (Greenbaum et al., 2016; Ito et al., 2019; Davin et al., 2010; Simonetti, 2011) Beyond aHUS, eculizumab has been explored for other forms of thrombotic microangiopathy (TMA). Studies have reported favorable responses in adult and pediatric HSCT-recipients with complement-mediated TA-TMA (Dvorak et al., 2019; Zhang et al., 2021; Jodele et al., 2020; de Fontbrune et al., 2015; Dhakal et al., 2017). Growing evidence, including a prospective trial, supports eculizumab’s effectiveness in pediatric TA-TMA, improving survival and organ recovery (Jodele et al., 2024). Recent dosing strategies have further optimized its use, reinforcing its emerging role as a standard therapeutic option (Jodele et al., 2014; Jodele et al., 2020; Jodele et al., 2024). Additionally, retrospective studies have described both favorable and controversial outcomes in pediatric patients with STEC-HUS treated with eculizumab (Monet-Didailler et al., 2020; Walsh and Johnson, 2019; Percheron et al., 2018; Garnier et al., 2023). STEC-HUS patients should be considered eligible for eculizumab treatment based on clinical severity, TMA criteria, and the risk of multi-system involvement, as its use is reserved for cases where supportive care alone is insufficient. The studies evaluating the effectiveness and safety of eculizumab in aHUS (approved indication), as well as in STEC-HUS and TA-TMA (off-label use), have used different outcome measures, making direct comparisons challenging. Additionally, the small pediatric sample sizes in these studies further limit generalizability.

Due to the overlapping presentation and clinical features, patients diagnosed with complement HUS, STEC-HUS, or TA-TMA are currently treated by the same clinicians and medical specialists. To evaluate the effectiveness of eculizumab in these types of TMA, homogeneous clinical measurements are essential.

The objective of this study was to assess the efficacy and safety of eculizumab in pediatric patients diagnosed with aHUS, STEC-HUS, and TA-TMA, using the same outcome measures, at a tertiary center in a real-world setting.

## Methods

Study design and patients: An observational retrospective study was conducted of all the patients diagnosed with aHUS, STEC-HUS, or TA-TMA at Schneider Children’s Medical Center during 2011–2020. Only the patients who received at least one dose of eculizumab for TMA treatment were included.

Demographic and baseline clinical data were collected, including HUS diagnosis and classification, the involvement of other organ systems, complement gene mutation status (if available), and prophylactic treatment for meningitis (vaccine and/or prophylactic amoxicillin) before eculizumab initiation.

Baseline and follow-up clinical parameters were documented. Hematologic parameters included platelet (PLT) count, lactate dehydrogenase (LDH) level and hemoglobin (Hb) concentration. Renal function was assessed according to the estimated glomerular filtration rate (eGFR), calculated by the bedside Schwartz equation, and the chronic kidney disease (CKD) stage, categorized according to the Kidney Disease: Improving Global Outcomes (KDIGO) 2021 clinical practice guideline (Kidney Disease: Improving Global Outcomes KDIGO Blood Pressure Work Group, 2021). Additionally, the need for dialysis or plasma infusion/plasma exchange (PI/PE) and CH-50 before or during follow-up was recorded. Data collection spanned a 28-week period following the first dose of eculizumab. For patients with a follow-up duration shorter than 28 weeks, data collection continued for 12 weeks after eculizumab discontinuation.

During the study period, eculizumab was administered according to body weight-based dosing recommendations provided by the manufacturer with adjustments required in administration frequency based on clinical and laboratory parameters and infused over 1–4 h (detailed in Supplementary Table S1).

Assessments of effectiveness: The primary endpoint was the proportion of patients who achieved a complete TMA response. This was defined as both sustained (≥4 weeks) normalization of hematologic parameters and improvement in renal function (detailed in Table 1) on two consecutive measurements.

**TABLE 1:** Definitions of the outcomes.

Outcome	Definition
TMA outcomes	
Complete TMA response (Primary end point)	Improvement in hematologic normalization and renal function was maintained for ≥4 weeks
TMA event free status	Platelet count did not decrease by >25% from baseline^a^, no PI/PE, and no new dialysis during ≥12 weeks
Hematologic outcome	Maintained for ≥4 weeks
Hematologic normalization	PLT count and LDH normalization
PLT count normalization	PLT count ≥150 K/μL
LDH normalization	LDH levels ≤ ULN according to age
Hemoglobin improvement	≥2 g/dL increase from baseline^a^
Renal outcome	Maintained for ≥4 weeks
eGFR improvement	≥15 mL/min/1.73 m^2^ increase from baseline^a^
CKD improvement	≥1 stage according to KDIGO classification (Kidney Disease: Improving Global Outcomes KDIGO Blood Pressure Work Group, 2021)
Renal function improvement	≥25% decrease in Cr_s_ from baseline^a^

^a^ Baseline refers to the level measured on the day of eculizumab initiation.

Abbreviations: CKD, chronic kidney disease; Cr, serum creatinine; eGFR, estimated glomerular filtration rate; KDIGO, kidney disease improving global outcomes; LDH, lactate dehydrogenase; PI/PE, plasma infusion/plasma exchange; PLT, platelets; TMA, thrombotic microangiopathy; ULN, upper limit of normal.

A secondary endpoint was TMA event-free status, which was defined by the absence of a PLT decreases by >25% from baseline as measured on the day of eculizumab initiation, the absence of PE/PI, and no new dialysis for ≥12 weeks. Other secondary endpoints comprised additional hematologic and renal improvements, including normalization of PLT, LDH, Hb, eGFR, and CKD stage, sustained for ≥4 weeks on two consecutive measurements (detailed in Table 1). The time to normalization of these parameters was calculated from the first eculizumab dose to the day that the first normal measurement was obtained. To avoid biased positive results, analyses of individual parameters: PLT, LDH, Hb, eGFR, and CKD excluded patients with normal baseline values, for whom the drug is expected to be ineffective.

Assessments of safety: Data on adverse events, changes in medical conditions, and causality were obtained from electronic medical records.

Statistical analysis: The study was approved by the Institutional Helsinki Committee of Schneider Children’s Medical Center, with a waiver for informed consent. Baseline characteristics are presented as means, medians, standard deviations (SDs), and ranges (for continuous variables); and as frequencies and proportions (for categorical variables). Mean changes (with 95% confidence intervals) from baseline in eGFR, Hb, LDH, and PLT were calculated over 28 weeks. Two patients with end-stage kidney disease (ESKD) were excluded from the eGFR analysis. The data was assessed according to the last observation carried forward (LOCF). The analyses were performed with IBM-SPSS version 26. Locally estimated scatter plot smoothing (LOESS) was performed to generate smoothed plots over the follow-up period, using the geom, smooth function in the ggplot2 package in R.

## Results

Twenty-five children and adolescents were recruited for the study. One of them was excluded from the analysis due to the absence of baseline data.

### Demographic characteristics

Patient demographics are summarized in Table 2. The majority (67%) were male. The median age was 5.8 years (range 0.1–21.9 years) at the initiation of eculizumab treatment. Six patients (25%) were younger than 2 years. The median body weight was 14.5 kg (range 5.1–51.3 kg). Thirteen children (54%) were diagnosed with aHUS, five (21%) with STEC-HUS, and six (25%) with TA-TMA. All six patients with TA-TMA had a prior diagnosis of graft versus host disease (GVHD). Seven oncological diagnoses were identified according to the International Classification of Diseases, 10th edition (Table 2). DNA sequencing of complement genes was performed in eight of the 13 children with aHUS. Three of them (38%) harbored genetic variants: one each with a MCP mutation, homozygous CFH mutation, and heterozygous CFH mutation. Genetic testing was not performed in the remaining five patients.

**TABLE 2:** Baseline demographics and disease characteristics of the study population (n = 24).

Age	
Median age at the first eculizumab dose, years, n = 24	5.8 (0.1–21.9)
1 month - <23 months, n	6 (25%)
≥23 months - <5 years, n	6 (25%)
≥5 years - <12 years, n	9 (38%)
≥12 years - <18 years, n	2 (8%)
≥18 years, n	1 (4%)
Weight	
Median weight, kg, n = 24	14.5 (5.1–51.3)
Sex	
Female, n	8 (33%)
Ethnicity, n	
Israeli Jewish	14 (58%)
Arab (Muslim)	5 (21%)
Others	5 (21%)
Diagnosis, n	
aHUS	13 (54%)
STEC-HUS	5 (21%)
TA-TMA	6 (25%)
Oncologic diagnosis	
Myeloid leukemia, n	2 (29%)
Myelodysplastic syndromes, n	2 (29%)
Hodgkin lymphoma, n	1 (14%)
Aplastic anemia, n	2 (29%)
Complement gene mutation among the 8 aHUS patients tested, n (%)/examined	3 (38%)
MCP	1
CFH – homozygous	1
CFH – heterozygous	1
no mutation found	5 (62%)
Median days from the first TMA symptom to the first eculizumab dose, n = 22	12.5 (1–2,805)
Median days from diagnosis to the first eculizumab dose, n = 22	2.5 (0–2,911)
Dialysis at diagnosis (past 1 year), n	14 (58%)
Mean platelet count, K/µL, (SD) n = 24	84 (89)
Platelet count <150 K/μL, n	19 (79%)
Mean LDH level, IU/L (SD), n = 24	3,359 (4,554)
LDH greater than ULN according to age, n	21 (88%)
Mean hemoglobin concentration, g/dL (SD), n = 24	8.6 (1.49)
Hemoglobin concentration <10 g/dL, n	20 (83%)
Schistocytes positive, n	23 (96%)
Mean eGFR, mL/min/1.73 m^2^ (SD), n = 24	43 (59)
eGFR, (mL/min/1.73 m^2^), n	24
<15, n	10 (42%)
15–29, n	5 (21%)
30–44, n	3 (13%)
45–59, n	1 (4%)
60–89, n	2 (8%)
>90, n	3 (13%)
Median duration of eculizumab treatment, weeks, n = 24	9.21 (0–82.0)
<1 week, n	4 (17%)
≥1 week, <4 weeks, n	3 (13%)
≥4 weeks, <28 weeks, n	12 (50%)
≥28 weeks, n	5 (21%)

The data are presented as median (range) or as number (%).

Abbreviations: aHUS, atypical hemolytic uremic syndrome; CFH, complement factor H; eGFR, estimated glomerular filtration rate; LDH, lactate dehydrogenase; MCP, membrane-cofactor protein; STEC-HUS, Shiga toxin-producing *E. coli* HUS; TA-TMA, transplant-associated thrombotic microangiopathy; TMA, thrombotic microangiopathy; ULN, upper limit of normal.

Prior to treatment with eculizumab, 19 children (79%) had eGFR below 60 mL/min/1.73 m^2^; 10 (42%) had eGFR under 15 mL/min/1.73 m^2^. Dialysis was implemented in 14 (58%) at diagnosis, and 3 (13%) aHUS patients received plasmapheresis during the period before eculizumab treatment was available, thus reflecting standard management practices. Prophylactic antibiotic therapy was administered to all the patients; 46% (n = 11) received a meningococcal vaccination before or immediately after the first eculizumab dose. Of the 13 patients who were not vaccinated, six were diagnosed with TA-TMA and GVHD due to the established inefficacy of vaccination during the immediate post-transplant phase. One patient was below the age threshold for vaccination. The remaining six patients, diagnosed with aHUS and STEC-HUS, required immediate eculizumab intervention, received prophylactic antibiotics, tailored to their individual clinical risk assessment, throughout their eculizumab treatment period. Vaccination was planned to be administered later during therapy, in accordance with established guidelines.

### Clinical endpoints of effectiveness and improvement over time

**FIGURE 1:**
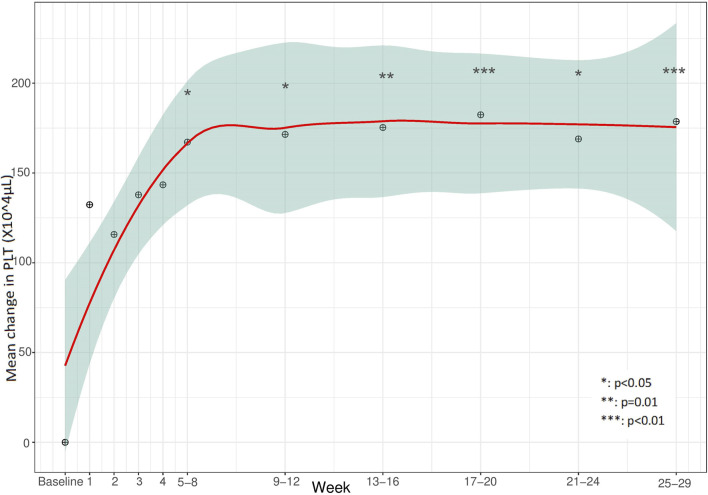
Mean change in platelets (PLT) during the 28 weeks from baseline.

**FIGURE 2:**
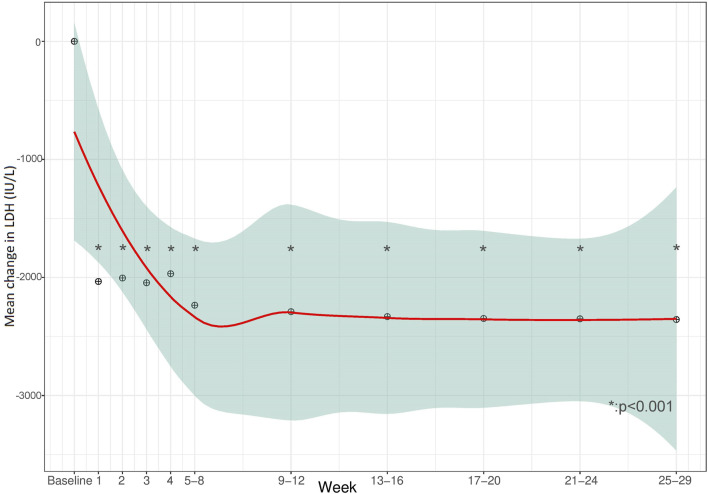
Mean change in lactate dehydrogenase levels (LDH) during the 28 weeks from baseline.

**FIGURE 3:**
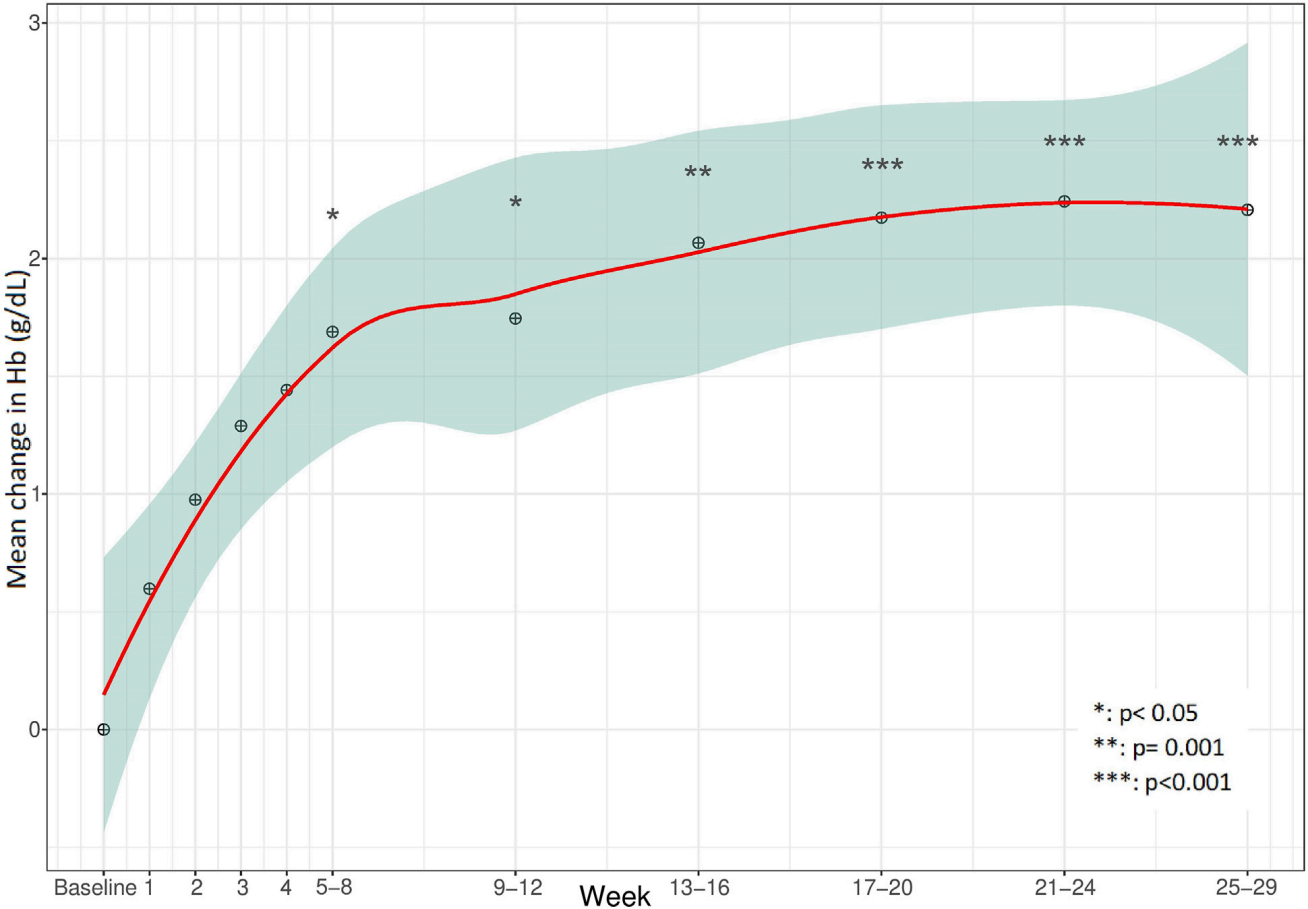
Mean change in hemoglobin (Hb) during the 28 weeks from baseline.

**FIGURE 4:**
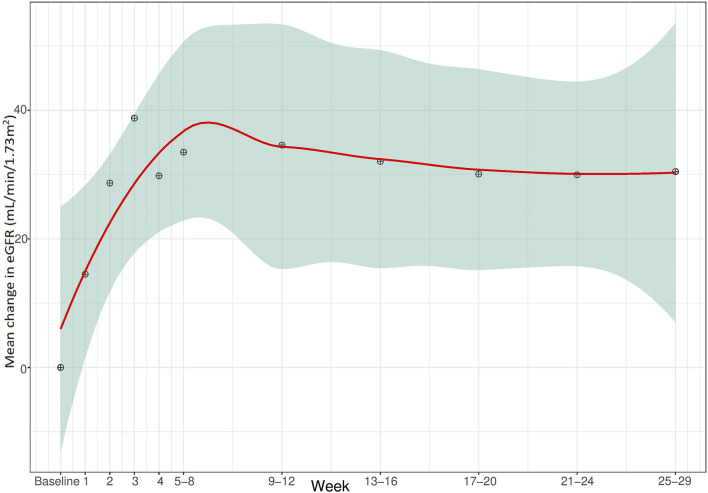
Mean change in eGFR during the 28 weeks from baseline.

Clinical endpoints of effectiveness during eculizumab treatment are presented in Table 3. Complete TMA response, TMA event-free status, and hematologic normalization (defined in Table 1) were achieved in 12/24 patients (50%, 95% CI 31.4%–68.6%), 15/24 patients (63%, 95% CI 42.7%–78.8%) and 16/24 patients (67%, 95% CI 46.7%–82.0%), respectively. Detailed results of endpoint achievement per patient in each TMA subtype are presented in Supplementary Tables S2–4. To avoid biased positive results, normalization of PLT count, LDH levels, Hb levels, and improvement in eGFR and CKD, each separately, included children whose analyzed parameter was abnormal prior to eculizumab initiation, and for whom effectiveness of the drug was expected. Children with baseline values within the target are already considered to be normalized, and therefore not included in the total number of some secondary clinical endpoints. Accordingly, Figures 1–4 demonstrate the mean change (95% CI) from baseline in laboratory clinical outcomes, achieved by the patients who were expected to improve.

**TABLE 3:** Clinical endpoints of effectiveness.

Indication	All study cohort	aHUS	STEC-HUS	TA-TMA
TMA outcomes				
Complete TMA response, n	24	13	5	6
n	12 (50%)	7 (54%)	3 (60%)	2 (33%)
95% CI	31.4–68.6	29.1–76.8	23.1–88.2	9.7–70.0
TMA event-free status, n	24	13	5	6
n	15 (63%)	9 (69%)	3 (60%)	3 (50%)
95% CI	42.7–78.8	42.4–87.3	23.1–88.2	18.8–81.2
Hematologic outcome				
Hematologic normalization, n	24	13	5	6
n	16 (67%)	9 (69%)	4 (80%)	3 (50%)
95% CI	46.7–82.0	42.4–87.3	37.6–96.4	18.8–81.2
PLT count normalization, n	19	9	4	6
n	12 (63%)	6 (67%)	3 (75%)	3 (50%)
95% CI	41.0–80.9	35.4–87.9	30.1–95.4	18.8–81.2
LDH normalization, n	21	11	4	6
n	16 (76%)	9 (82%)	3 (75%)	4 (67%)
95% CI	54.9–89.4	52.3–94.9	30.1–95.4	35.4–87.9
Hemoglobin improvement ≥2 g/dL, n	20	10	5	5
n	12 (60%)	6 (60%)	3 (60%)	3 (60%)
95% CI	38.7–78.1	31.3–83.2	23.1–88.2	23.1–88.2
Renal outcome				
eGFR improve by ≥ 15 mL/min/1.73 m^2^, n	19	11	5	3
n	15 (79%)	9 (82%)	4 (80%)	2 (67%)
95% CI	54.4–93.9	48.2–97.7	28.4–99.5	9.4–99.2
CKD improvement by ≥ 1 stage, n	19	11	5	3
n	17 (90%)	9 (82%)	5 (100%)	3 (100%)
95% CI	66.9–98.7	48.2–97.7	47.8–100.0	29.2–100.0
The mean number of days that the first dose of eculizumab followed		10	5	6
The first TMA symptom		4.5	16	79
The diagnosis of HUS		1.5	8	1.5

Abbreviations: aHUS, atypical hemolytic uremic syndrome; CKD, chronic kidney disease; eGFR, estimated glomerular filtration rate; HUS, hemolytic uremic syndrome; LDH, lactate dehydrogenase; PLT, platelets; STEC-HUS, Shiga toxin-producing *E. coli* HUS; TA-TMA, transplant-associated thrombotic microangiopathy; TMA, thrombotic microangiopathy.

The mean PLT count was 84 K/μL (SD 89 K/μL, 8–300 K/μL) at baseline for all 24 patients included in the cohort. The PLT count improved significantly within 5–8 weeks for 12/19 (63%, 95% CI 41.0%–80.9%) patients with baseline abnormality, after a mean 37.3 days (SD 42.5 days) (Figure 1).

The trends observed for LDH were similar to those for PLT. The mean LDH count was 3359 IU/L (SD 4554 IU/L, 364–21657 IU/L (at baseline for all 24 patients included in the cohort. Improvement was significant within the first week for 16/21 (76%, 95% CI 54.9%–89.4%) children with baseline abnormality, after a mean 20.6 days (SD 18.4 days) (Figure 2).

The mean Hb count was 8.6 g/dL (SD 1.49 g/dL, 6.1–11.7 g/dL (at baseline for all 24 patients included in the cohort. Hb improved significantly (by ≥ 2 g/dL) within 5–8 weeks, for 12/20 (60%, CI 95% 38.7%–78.1%) of the children with baseline abnormality, after a mean 69.5 days (SD 95.51 days) (Figure 3).

The mean eGFR was 43 mL/min/1.73 m^2^ (SD 59 mL/min/1.73 m^2^, 4–242 mL/min/1.73 m^2^) at baseline for all 24 patients included in the cohort. However, renal outcomes analysis excluded five children: three with normal eGFR levels, and two with focal segmental glomerular sclerosis on dialysis. eGFR improved by ≥ 15 ml/min/1.73 m^2^ in 15/19 patients (79%, 95% CI 54.4%–93.9%) after a mean 69 days (SD 168.3 days); this change from baseline was not statistically significant (Figure 4). CKD stage improved in 17/19 patients (90%, 95% CI 66.9%–98.7%).

### Clinical endpoints of effectiveness according to TMA subtype

Table 3 presents the clinical endpoints of effectiveness, according to indications, during eculizumab treatment. The primary outcome, a complete TMA response, was achieved in 54% (95% CI 29.1%–76.8%) of the patients with aHUS, 60% (95% CI 23.1%–88.2%) of those with STEC-HUS, and 33% (95% CI 9.7%–70.0%) of those with TA-TMA.

Secondary outcomes were analyzed only in children with abnormal baseline values and were achieved to varied extents, as detailed in Table 3. Eculizumab treatment yielded positive renal outcomes. Among patients with renal impairment (n = 19), an increase in eGFR of ≥ 15 mL/min/1.73 m^2^ was observed in 82% (95% CI 48.2%–97.7%) of those with aHUS, 80% (95% CI 28.4%–99.5%) of those with STEC-HUS, and 67% (95% CI 9.4%–99.2%) of those with TA-TMA. Additionally, improvement in at least one CKD stage was achieved in most patients (Table 3). Of the 14 patients on dialysis at eculizumab initiation, five (three with aHUS, two with STEC-HUS) remained dialysis-dependent post-treatment: One aHUS patient had pre-existing ESKD due to FSGS and was dialysis-dependent before aHUS onset. One aHUS patient deceased shortly after only one dialysis course. Long-term dialysis status was uncertain for two patients (one aHUS, one STEC-HUS) due to loss of follow-up after transfer to other institutions, and a STEC-HUS patient that stopped dialysis 4 days after only one eculizumab dose.

While patients with aHUS and STEC-HUS were treated with the standard aHUS-based eculizumab regimen. TA-TMA patients received an individualized intensified dosing regimen, with initial doses administered every 3–7 days, followed by maintenance dosing every 14 days upon clinical and laboratory stabilization.

All TA-TMA patients developed severe GVHD (stage 3–4) before diagnosis, with a median onset of 3.5 weeks (range: 1–10 weeks). Gastrointestinal GVHD was present in 5 patients, hepatic GVHD in 4, and skin GVHD in 3, with 4 patients having more than one type. Outcomes varied: 2 patients had complete GVHD resolution, 3 had partial resolution with chronic GVHD, and 1 had unresolved GVHD. Two patients died from infection-related complications.

The mean CH-50 level obtained in nine children before the start of eculizumab treatment was 67% (SD 20.18%). As expected, a significantly lower measurement, of 14% (SD 21%), was achieved after treatment initiation, in 13 children (t-test, p < 0.001).

Paired data were available for six children, for whom measurements were taken for this outcome, both before and after treatment initiation. The mean CH-50 level was lower after than before treatment initiation: 6% (SE 3.59) vs. 69% (SE 5.99), p = 0.001 (t-test).

### Time to initiate eculizumab, from diagnosis or from the first symptom

Three aHUS patients were excluded from this analysis: one due to missing medical records (excluded from Tables 2, 3) and two who were diagnosed before eculizumab approval (excluded from Table 3 analysis). Both of these pre-approval patients were successfully treated with eculizumab, meeting all clinical outcomes.

Six patients experienced a delay of more than 30 days between the first TMA symptoms and eculizumab initiation (Tables 2, 3) of them only one aHUS patient initiated the treatment more than 30 days from diagnosis. In four TA-TMA patients, diagnostic uncertainty contributed to the delay, as anemia, thrombocytopenia, hypertension, and proteinuria, which are hallmarks of TA-TMA, also occur in GVHD; as well as infections, and drug toxicity may also complicate early recognition. No TA-TMA patient initiated treatment more than 30 days from diagnosis. The remaining two patients had unique challenges. One had FSGS, chronic dialysis, and hypertension, making it difficult to confirm aHUS as the cause of TMA, delaying treatment decisions. The other had been diagnosed with aHUS several years prior, when eculizumab was not yet approved, and received treatment only later through a compassionate-use program.

Eculizumab was administered to three children with TA-TMA who had normal kidney function, in an attempt to improve their hematological status. The two children who died at 9- and 21-day following treatment initiation received only one and five doses, respectively. Thus, they did not meet the required duration for achieving the clinical endpoints. However, the remaining four children exhibited improvements after a minimum of 5 weeks of treatment, with a mean of 10.5 (ranging from 6 to 14) doses of eculizumab.

Eculizumab was discontinued in patients with STEC-HUS and TA-TMA primarily due to clinical improvement, reaching clinical outcomes, including renal recovery and hematologic normalization. In four patients (two aHUS, two TA-TMA) treatment was discontinued due to patients’ death. Among the 11 surviving aHUS patients, reasons for cessation varied: two medical tourists returned to their home counties post-optimization, four (including two with CFH mutations) transitioned to other institutions, and five discontinued after sustained clinical improvement—three with pre-existing conditions (FSGS-related ESKD or antiphospholipid syndrome) and two additional patients (one with MCP genetic mutation) discontinued treatment after achieving sustained clinical improvement without relapse during follow-up period. No discontinuations occurred due to drug unavailability, safety concerns, or adverse events.

### Safety of patients

The study population (n = 24) consisted of critically ill children with TMA, sometimes with multisystem involvement. A retrospective review of the medical records revealed that no clinical or laboratory manifestation could be attributed to eculizumab treatment based on the side effects reported in the literature. Four of the 24 patients included in the study (17%, 95% CI 5.0%–38.8%) died. Two of them were diagnosed with TA-TMA and the other two with aHUS induced by chemotherapy. All the deaths were attributed to the children’s underlying disease and infection-related complications due to bone marrow transplantation, regardless of the use of eculizumab.

## Discussion

In this retrospective study of 24 patients treated with eculizumab for HUS at one tertiary medical center, efficacy was demonstrated overall, and no severe adverse events were attributed to the drug. This study contributes to the existing literature by evaluating eculizumab across three TMA subtypes using consistent outcome measures.

The clinical outcomes we report for aHUS are consistent with the previously published results (Greenbaum et al., 2016; Ito et al., 2019), supporting the use of eculizumab as a first-line treatment in pediatric populations. Complete TMA response, the primary outcome, was achieved in 54% of aHUS patients, comparable to rates in pediatric reports of 64% by Greenbaum et al., (2016) and 36% by Ito et al., (2019) adult studies (Fakhouri et al., 2016). A significant proportion of patients also achieved TMA event-free status, hematologic normalization, and renal improvement, aligning with previously reported high success rates (Greenbaum et al., 2016; Ito et al., 2019).

Of our six patients with aHUS who did not achieve a complete response, two (15%) had pre-existing ESKD unrelated to HUS and had been on dialysis for several years. Despite their lack of improvement in renal function during the follow-up period, these patients were included in the analysis, unlike previous studies (Greenbaum et al., 2016). Additionally, this allowed us to assess eculizumab’s impact on hematologic and other parameters in ESKD patients, though including children with pre-existing ESKD from non-HUS glomerular diseases inevitably biased this outcome.

In our study, 61.5% of aHUS patients underwent genetic testing, which aligns with reported rates in retrospective studies (Ito et al., 2019) and is comparable to some prospective studies (Licht et al., 2015; Greenbaum et al., 2016) where systematic genetic screening was not mandated. While genetic testing provides valuable insights into complement dysregulation, it is not required for diagnosing aHUS or initiating eculizumab treatment (Licht et al., 2015; Zuber et al., 2012; Campistol et al., 2013). Given that results may take months to obtain, clinicians often rely on clinical presentation rather than genetic findings to guide immediate management (Noris et al., 2010; Licht et al., 2015; Fremeaux-Bacchi et al., 2013; Zuber et al., 2012). Our findings highlight that, in real-world settings, genetic testing, is not prioritized during acute conditions and performed selectively based on clinical judgment, rather than as a universal requirement.

STEC-HUS patients in our study were evaluated using the same criteria as aHUS patients, with 60% achieving complete TMA response and TMA event-free status, and all showing renal function improvement. Our study provides additional data on eculizumab use in pediatric STEC-HUS, including cases with and without neurological or systemic involvement (60% and 40%, respectively). Both subgroups showed clinical improvement, though not all met predefined outcomes due to rigorous criteria, the notable clinical advancements and highlight eculizumab potential benefit.

Eculizumab treatment was initiated based on clinical severity, including TMA diagnostic criteria and the risk mentioned of multi-system involvement, particularly neurological or cardiovascular complications. Since eculizumab is not ultimately indicated for STEC-HUS, its use was reserved for cases where supportive care alone was insufficient. This selection approach aligns with current evidence, which suggests that while complement activation plays a role in STEC-HUS pathogenesis, its clinical significance varies (Garnier et al., 2023), necessitating individualized treatment decisions.

Several studies have investigated the effectiveness of eculizumab in treating STEC-HUS, highlighting both benefits and limitations. Retrospective studies by Giordano et al., (2019) and Percheron et al., (2018) underscored the importance of early treatment, particularly when the presentation is severe and with neurological involvement, showing improvement in hematologic and neurological outcomes. Percheron et al. also suggested that sustained complement inhibition with eculizumab might correlate with favorable outcomes.

However, findings on eculizumab’s effectiveness in milder STEC-HUS cases are mixed. Monet-Didailler et al. (2020) reported no significant differences in blood pressure, proteinuria, or renal function between eculizumab-treated and untreated patients at 1- and 12-months follow-ups, suggesting limited efficacy in mild cases. Garnier et al. (2023) randomized controlled trial showed no difference in acute disease progression between eculizumab-treated and control groups, but observed less renal damage after 1 year in the treatment group. These findings indicate that eculizumab may be effective in severe presentations, as supported by our study’s results, but its benefits in milder presentations remain uncertain, reinforcing the need for careful patient selection.

This study adds to the growing evidence supporting eculizumab’s effectiveness in treating TA-TMA in children post-bone marrow transplant. Consistency across studies strengthens our understanding of eculizumab’s efficacy in managing TA-TMA. Jodele et al. (2020) and Jodele et al. (2020), Dhakal et al., 2017) reported improved clinical outcomes in 66% and 92% of surviving children, respectively, 1 year after adding eculizumab to other treatments. More recently, a prospective study by Jodele et al. (2024) in children with multi-organ injury demonstrated a 71% survival rate at 6 months and 62% at 1 year, further reinforcing eculizumab’s role in high-risk TA-TMA (Jodele et al., 2024). In comparison, our study observed a 67% survival rate, which aligns with these previous findings. Additionally, 50% of our TA-TMA patients achieved TMA event-free status and hematological normalization, while 33% attained complete TMA response. Our study employed stricter outcome definitions, requiring multiple hematologic and renal criteria for response, in contrast to prior studies that primarily assessed survival. This difference in outcome measures may account for variations in reported response rates. Renal function improved in the three relevant children, further supporting eculizumab as a promising treatment for children with TA-TMA and preserved kidney function, though larger studies are needed. Notably, during our study period, and still today, a standardized, consensus-based regimen for TA-TMA remains under investigation with precision-guided dosing strategies only recently emerging to further optimize eculizumab’s effectiveness (Jodele et al., 2024; Mizuno et al., 2022). Recognizing the need for intensified dosing regimen in TA-TMA, as supported by recent studies (Jodele et al., 2024; Mizuno et al., 2022), an individualized dosing approach was adapted with intensified frequent initial dosing, guided by clinical and laboratory biomarkers to optimize outcomes.

Like prior research, our study focused on children with severe TA-TMA manifestations. Notably, all six TA-TMA patients in our cohort had pre-eculizumab GVHD, a factor associated with increased non-relapse mortality and high-risk TA-TMA, as described in previous studies (de Fontbrune et al., 2015; Schoettler et al., 2023). The high severity of GVHD and the two infection-related deaths in our cohort further underscore the strong temporal and clinical link between severe GVHD and TA-TMA, with GVHD typically preceding TA-TMA by several weeks. However, prognosis is multifactorial, with complement activation markers, multi-organ dysfunction, and infections also play a critical role (Schoettler et al., 2023). Further research is needed to assess eculizumab’s efficacy in milder disease and across a broader spectrum of TA-TMA severity.

The recently introduced TA-TMA risk stratification system (Schoettler et al., 2023; Carabante et al., 2024) classifies all six patients in our cohort as ‘High-Risk TMA’. This was based on severe GVHD (Grade 3–4) and peak LDH >2 times ULN in all patients, in addition to systemic infections, random urine protein-to-creatinine ratio (rUPCR) ≥1 mg/mg, and organ dysfunction (excluding acute kidney injury) in some of them. Given the poorer prognosis associated with high-risk TA-TMA, this classification helps contextualize our cohort’s response rates.

Diagnosing TA-TMA remains challenging due to overlapping of non-specific symptoms with common post-HSCT complications such as GVHD, infections, and drug toxicities (Schoettler et al., 2023) and lacks specific biomarkers. These share clinical features such as anemia, thrombocytopenia, hypertension, and proteinuria that often delay recognition and, consequently, eculizumab initiation. Additionally, the lack of standardized TA-TMA screening and treatment protocols—which have only recently emerged in clinical practice—has further contributed to treatment delays (Mizuno et al., 2022). Earlier initiation of eculizumab has been suggested to improve clinical outcomes (de Fontbrune et al., 2015), emphasizing the importance of timely diagnosis.

The delayed initiation of eculizumab in four TA-TMA patients was attributed to these diagnostic challenges. Beyond TA-TMA cases, two additional aHUS patients faced delays in eculizumab initiation due to unique challenges. One patient, with a history of FSGS leading to chronic dialysis and hypertension, had overlapping symptoms, making it difficult to confirm aHUS as the cause of TMA and delaying treatment. Another patient had been diagnosed with aHUS for several years before eculizumab was approved, treated with plasmapheresis and received treatment through a compassionate-use program. Remarkably, only one aHUS patient in our cohort initiated treatment more than 30 days from diagnosis, and no TA-TMA patient experienced such a delay.

Notably, some of our patients with both aHUS and STEC-HUS had favorable outcomes after prompt administration of eculizumab. Our findings collaborate those (Goodship et al., 2017; Dhakal et al., 2017; Giordano et al., 2019; Loirat et al., 2016) who claimed that the timing of eculizumab initiation relative to symptom onset and the involvement of additional organ systems are key factors influencing treatment efficacy in children.

Compared to single-timepoint measurements, our longitudinal monitoring of PLT, LDH, Hb, and eGFR over 28 weeks provided a more comprehensive evaluation of treatment outcomes and better informing of clinical decision-making. The use of the LOCF method helped overcome the challenges due to variations in follow-up durations and measurement frequency. The most significant changes occurred within the first 5 weeks of treatment. Statistically significant improvement was observed in all the parameters from baseline onwards, except for eGFR, for which a trend of improvement was exhibited. This observation can be attributed to the inclusion of two children with ESKD requiring dialysis (2/24) and three with normal baseline eGFR (3/24). Given their pre-existing renal status, these five children, representing 21% of the cohort, were not expected to show any improvement in their renal function. Consequently, their inclusion may have masked the overall improvement in eGFR. However, the remaining 19 children displayed notable improvements in eGFR and other renal parameters, underscoring the positive impact of eculizumab treatment on renal function in the majority of the cohort.

As expected, CH-50 assay values decreased significantly after eculizumab initiation, potentially reaching complete absence of activity of the complement system, thus confirming the drug’s effect. As a measure of complement system activity and an indication of the extent of MAC blockade by eculizumab, the CH-50 assay reflects treatment efficacy and aids in dosage adjustments, especially in patients with TA-TMA and high complement activation (Jodele et al., 2020).

### Safety

No adverse events or worsening of the medical condition were attributed to eculizumab. However, due to the complexities of the disease states and comorbidities, attributing symptoms to treatment is challenging. In addition, the retrospective design relied on varied documentation. As some treatments were administered in outpatient clinic settings, symptoms from previous administrations may have been missed. Moreover, common, non-specific adverse events such as headache, hypertension, diarrhea, or nausea and vomiting (Soliris Eculizumab, 2019; ECULIZUMAB, 2021a; ECULIZUMAB, 2021b) further complicate attributing them to the drug treatment.

Four of 24 children died: two aHUS patients secondary to complications of chemotherapy and two TA-TMA patients due to severe infections. This underscores infection as a key mortality factor in TA-TMA, unlike primary HUS, where severe multi-organ impairment predominates. No deaths were attributed to eculizumab.

### Limitations

This study has several limitations. This was a retrospective observational study in its nature, relying on data extracted from medical records, performed by several physicians and according to their discretion, not according to a predefined protocol. This resulted in inconsistencies, which posed challenges for data collection and verification. Furthermore, complete 28-week data, the ideal duration, was unavailable for all the children, especially those who died or experienced rapid treatment response. To address missing data points, we employed the LOCF statistical method.

In addition, Data collection (2011–2020) varied due to evolving documentation and record systems, affecting data uniformity. We also acknowledge that the small sample size, lack of a control group, and limited sub-group sizes also hindered definitive conclusions for each indication.

Another limitation of this retrospective study is the selective use of genetic testing (61.5% of aHUS patients) based on clinical judgment rather than a standardized protocol, reflecting real-world practice in our and comparable studies and may limit genetic characterization (Licht et al., 2015; Greenbaum et al., 2016; Ito et al., 2019). Additionally, detailed information on the exact location of CFH and MCP mutations were unavailable consistent with smilar prospective and retrospective studies (Licht et al., 2015; Ito et al., 2019; Fakhouri et al., 2016).

## Conclusion

Using the same standardized parameters for evaluating pediatric patients with aHUS, STEC-HUS, and TA-TMA, eculizumab was shown to be efficacious and safe. Overall, hematological and renal parameters improved, and the need for plasmapheresis and dialysis decreased. TMA progression was prevented in children with complement-mediated HUS. Moreover, early intervention was more effective across all HUS indications. The unique inclusion of patients with ESKD highlights the drug’s beneficial effects beyond renal improvement, and its potential to impact additional organ systems. The diversity in symptom severity and system involvement, including the neurological system, provide a comprehensive analysis of eculizumab’s efficacy. Well-designed, prospective, randomized controlled trials are needed, encompassing a larger, more diverse patient population, with varying disease severities.

## Data Availability

The original contributions presented in the study are included in the article/Supplementary Material, further inquiries can be directed to the corresponding author.
