# Supplementary material for: Eculizumab is efficacious and safe in pediatric patients with various forms of hemolytic uremic syndrome: a retrospective clinical experience of a tertiary center
Source: Front Pharmacol. 2025 Apr 4;16:1535407. doi: 10.3389/fphar.2025.1535407 (PMC12006164; doi:10.3389/fphar.2025.1535407)
Supplement: Supplementary file 1 [file Table1.DOCX]

**Eculizumab is efficacious and safe in pediatric patients with different forms hemolytic uremic syndrome: A retrospective clinical experience of a tertiary center**

Supplemental Information

| Table S1- Schedule of eculizumab dose administration based on patient body weight | | |
| --- | --- | --- |
| **Maintenance** | **Induction** | **Patient Body Weight** |
| 1200 mg at week 5, then 1200 mg every 2 weeks | 900 mg weekly x 4 weeks | ≥40 kg |
| 900 mg at week 3, then 900 mg every 2 weeks | 600 mg weekly x 2 weeks | 30 to <40 kg |
| 600 mg at week 3, then 600 mg every 2 weeks | 600 mg weekly x 2 weeks | 20 to <30 kg |
| 300 mg at week 2, then 300 mg every 2 weeks | 600 mg weekly x 1 week | 10 to <20 kg |
| 300 mg at week 2, then 300 mg every 3 weeks | 300 mg weekly x 1 week | 5 to <10 kg |
| * Dosages may vary at medical discretion | | |

| Table S2- The efficacy of eculizumab treatment in patients diagnosed with aHUS | | | | | | | | |
| --- | --- | --- | --- | --- | --- | --- | --- | --- |
| Renal outcomes | | Hematologic outcomes | | | | TMA outcomes | | Patient number |
| CKD improvement | eGFR improvement | Hb improvement | LDH normalization | PLT count normalization | Hematologic normalization | TMA event free status | Complete TMA response |  |
| ------ | ------ | ✓ | - | - | ✓ | ✓ | 🗶 | 1 |
| ✓ | ✓ | 🗶 | ✓ | 🗶 | 🗶 | ✓ | 🗶 | 2 |
| ✓ | ✓ | ✓ | ✓ | ✓ | ✓ | ✓ | ✓ | 3 |
| ✓ | ✓ | 🗶 | ✓ | - | ✓ | ✓ | ✓ | 4 |
| ✓ | ✓ | ✓ | ✓ | ✓ | ✓ | ✓ | ✓ | 5 |
| ------ | ------ | ✓ | ✓ | ✓ | ✓ | ✓ | 🗶 | 6 |
| ✓ | ✓ | ✓ | ✓ | ✓ | ✓ | ✓ | ✓ | 7 |
| 🗶 | 🗶 | 🗶 | - | 🗶 | 🗶 | 🗶 | 🗶 | 8 |
| ✓ | ✓ | - | ✓ | - | ✓ | 🗶 | ✓ | 9 |
| ✓ | ✓ | 🗶 | 🗶 | ✓ | 🗶 | ✓ | 🗶 | 10 |
| ✓ | ✓ | - | ✓ | - | ✓ | 🗶 | ✓ | 11 |
| 🗶 | 🗶 | - | 🗶 | 🗶 | 🗶 | 🗶 | 🗶 | 12 |
| ✓ | ✓ | ✓ | ✓ | ✓ | ✓ | ✓ | ✓ | 13 |
| 82% | 82% | 60% | 82% | 67% | 69% | 69% | 54% | Current study (total) |
| Not tested | 75% | 63% | 48% | 78% | 41% | 85% | 36% | Ito et al. |
| 77% | 86% | 68% | 82% | 95% | 82% | 95% | 64% | Greenbaum et al. |
| Abbreviations: aHUS, atypical hemolytic uremic syndrome; CKD, chronic kidney disease; eGFR, estimated glomerular filtration rate; Hb, hemoglobin; LDH, lactate dehydrogenase; PLT, platelets; TMA, thrombotic microangiopathy. | | | | | | | | |

| Table S3- The efficacy of eculizumab treatment in patients diagnosed with STEC-HUS | | | | | | | | |
| --- | --- | --- | --- | --- | --- | --- | --- | --- |
| Renal outcomes | | Hematologic outcomes | | | | TMA outcomes | | Patient number |
| CKD improvement | eGFR improvement | Hb improvement | LDH normalization | PLT count normalization | Hematologic normalization | TMA event free status | Complete TMA response |  |
| ✓ | ✓ | ✓ | ✓ | ✓ | ✓ | ✓ | ✓ | 1 |
| ✓ | ✓ | 🗶 | - | ✓ | ✓ | ✓ | 🗶 | 2 |
| ✓ | ✓ | ✓ | ✓ | ✓ | ✓ | 🗶 | ✓ | 3 |
| ✓ | ✓ | ✓ | ✓ | - | ✓ | ✓ | ✓ | 4 |
| ✓ | 🗶 | 🗶 | 🗶 | 🗶 | 🗶 | 🗶 | 🗶 | 5 |
| **100%** | **80%** | **60%** | **75%** | **75%** | **80%** | **60%** | **60%** | Current study (total) |
| Abbreviations: CKD, chronic kidney disease; eGFR, Estimated glomerular filtration rate; Hb, hemoglobin; LDH, lactate dehydrogenase; PLT, Platelets; STEC-HUS, Shiga toxin-producing E. coli - hemolytic uremic syndrome; TMA, thrombotic microangiopathy | | | | | | | | |

| Table S4- The efficacy of eculizumab treatment in patients diagnosed with TA-TMA | | | | | | | | |
| --- | --- | --- | --- | --- | --- | --- | --- | --- |
| Renal outcomes | | Hematologic outcomes | | | | TMA outcomes | | Patient number |
| CKD improvement | eGFR improvement | Hb improvement | LDH normalization | PLT count normalization | Hematologic normalization | TMA event free status | Complete TMA response |  |
| - | - | ✓ | ✓ | ✓ | ✓ | ✓ | ✓ | 1 |
| - | - | 🗶 | 🗶 | 🗶 | 🗶 | 🗶 | 🗶 | 2 |
| - | - | - | 🗶 | 🗶 | 🗶 | 🗶 | 🗶 | 3 |
| ✓ | 🗶 | 🗶 | ✓ | ✓ | ✓ | 🗶 | 🗶 | 4 |
| ✓ | ✓ | ✓ | ✓ | ✓ | ✓ | ✓ | ✓ | 5 |
| ✓ | ✓ | ✓ | ✓ | 🗶 | 🗶 | ✓ | 🗶 | 6 |
| **100%** | **67%** | **60%** | **67%** | **50%** | **50%** | **50%** | **33%** | Current study (total) |
| Abbreviations: CKD, chronic kidney disease; eGFR, Estimated glomerular filtration rate; Hb, hemoglobin; LDH, lactate dehydrogenase; PLT, platelets; TA-TMA, transplant-associated thrombotic microangiopathy; TMA, thrombotic microangiopathy | | | | | | | | |
